# A Novel Numerical Procedure to Estimate the Electric Charge in the Pore from Filtration of Single-Salt Solutions

**DOI:** 10.3390/membranes11100726

**Published:** 2021-09-23

**Authors:** Patrick Dutournié, T. Jean Daou, Sébastien Déon

**Affiliations:** 1 Institut de Science des Matériaux de Mulhouse (IS2M-UMR CNRS 7361), Université de Haute-Alsace, 3Bis Rue Alfred Werner, CEDEX, 68098 Mulhouse, France; patrick.dutournie@uha.fr (P.D.); jean.daou@uha.fr (T.J.D.); 2 Institut UTINAM (UMR CNRS 6213), Université de Bourgogne Franche-Comté, 16 route de Gray, CEDEX, 25030 Besançon, France

**Keywords:** transport modelling, ceramic membranes, parameter assessment, membrane charge, adsorption isotherms

## Abstract

The assessment of physicochemical parameters governing the transport of ions through nanoporous membranes is a major challenge due to the difficulty in experimental estimation of the dielectric constant of the solution confined in nanopores and the volumetric membrane charge. Numerical identification by adjusting their values to fit experimental data is a potential solution, but this method is complicated for single-salt solutions due to the infinite number of couples that can describe a rejection curve. In this study, a novel procedure based on physical simplifications which allows the estimation of a range of values for these two parameters is proposed. It is shown here that the evolution of the interval of membrane charge with salt concentration can be described in all the experimental conditions by the Langmuir–Freundlich hybrid adsorption isotherm. Finally, it is highlighted that considering the mean dielectric constant and the adsorption isotherms assessed from a range of concentrations allowed a good prediction of rejection curves, irrespective of the salt and membrane considered.

## 1. Introduction

Pressure-driven membrane processes are often used to solve environmental issues such as desalination, wastewater treatment, drinking water production, etc. Among the potential applications, the removal of ionic contaminants is one of the main purposes for which membrane processes are particularly competitive due to the charged surface of commercial membranes. For decades, it has been well-know that the performances of nanoporous membranes (i.e., nanofiltration and low-molecular-weight cut-off ultrafiltration membranes) are governed by several physical complex mechanisms, and many research teams are still working on the development of accurate models which can describe and even predict the filtration performances of these membranes [1,2,3]. Many models are available in the literature, but a classical approach used by many researchers consists in considering an interfacial partitioning based on the Donnan equilibrium and steric exclusion, and describing the transport inside pores by the extended Nernst–Planck equation [4,5,6]. The vision of the transfer at the membrane/solution interfaces is usually improved by considering the dielectric exclusion due to a solvation energy barrier induced by changes in the dielectric constant of the solvent inside pores. This model, which was initially proposed by Bowen et al. [7], has proved a good ability to describe the experimental ion rejections obtained in many experimental conditions [8,9,10,11], provided that four parameters are known or adjusted. Two of these parameters are structural properties, whereas the two others are physicochemical properties of the membrane and the solution confined in nanopores. The two membrane structural parameters, namely intrinsic hydraulic permeability *L_p_* and mean pore radius *r_p_*, are easy to assess and are usually estimated from pure water flux and filtration of a neutral solute, respectively [12]. The estimation of the volumetric membrane charge *X_d_* and the dielectric constant of the solution inside pores *ε_p_* is more problematic and the way to assess them represents the key for a better understanding of transport mechanisms and the development of a predictive numerical tool. Indeed, these parameters are very complicated to assess experimentally, even if recent studies have shown that it is not completely unrealistic to develop original techniques to do so. For instance, streaming potential or current measurements [13,14,15] have demonstrated a high potential to determine membrane charge. However, only the estimation of the charge at the membrane surface is possible for nanoporous membranes (tangential measurements), and the charge at the pore wall remains undeterminable. Moreover, the presence of conduction and streaming current potentially occurring in membrane porosity also complicate the signal analysis. Electrochemical impedance spectroscopy [16,17,18] or membrane potential [19,20] are potential techniques to assess the dielectric constant of the solution inside pores, but the difficulty in separating the contribution of the solution to the signal of the wet membrane is often problematic. In any case, these methods are not completely satisfying at the present time and a numerical way is often required [21].

It has already been highlighted that there exists an infinite number of couples (*ε_p_*, *X_d_*) which allow the description of a salt rejection curve [22]. *ε_p_* and *X_d_*, since they have different impacts on ions, can have their values assessed from ion mixtures, for which only one couple can describe the various curves and be extrapolated to single-salt solutions [23]. However, this procedure, which has been presented and validated during previous studies, presents the inconvenience of requiring many experiments at various proportions before being extrapolated for single salts.

The simpler approach proposed in this paper thus consists of squeezing the values by physical deduction to reduce the interval of possible values that correctly describe single-salt rejections. In this paper, this procedure is implemented to determine the interval of membrane charge which allows a correct description of experimental curves. These intervals of charge are then computed to estimate the best adsorption isotherms and investigate the predictive ability of the proposed procedure in several experimental conditions of salts and membranes.

## 2. Materials and Methods

Experimental filtrations were performed with a laboratory pilot plant provided by TIA (Techniques Industrielles Appliquées, Bollène, France). Three ceramic membranes were used in this work, namely a TiO_2_ membrane (1 kDa) provided by TAMI Industries (Nyons, France) and two home-made zeolite membranes (Na-mordenite and a bi-layer MFI/MFI). The membranes (25 cm length with an inner diameter of 7 mm) consist of a tubular support of porous alumina, on which the active layer (titania or zeolite) was deposited. The specific preparation of zeolite membranes and their characteristics are detailed in previous papers [11,24,25].

The membrane module is mounted on the lab scale setup in stainless steel. The solution is introduced into the feed tank (5 L) and the fluid flow inside the membrane module is provided by a volumetric pump (piston, 2.2 kW with frequency variation, *Pmax* = 3 MPa) with a flow rate of 700 L/h (i.e., fluid velocity > 5 m/s). The applied pressure Δ*P* is adjusted from 4 to 12 bar by closing a manual valve. Experimental tests were conducted at a constant temperature of 25 °C (controlled by a cooling unit) and with a constant feed concentration by recycling both feed and retentate solutions.

First of all, the membrane is conditioned with pure water by filtrating demineralized water (conductivity < 0.1 µS/cm) to reach steady hydraulic performances. The filtration of an uncharged solute (vitamin B12 from Alfa Aesar, purity 98%, 9 × 10^−4^ mol m^−3^) was performed to investigate steric effect and estimate the mean pore radius of the membrane. Retentate and permeate streams were sampled and analysed by UV–visible spectrometry at 362 nm with a Lambda 35 spectrophotometer from Elmer Instrument, Waltham, USA.

Filtrations of single-salt solutions containing NaF, NaCl, NaI and Na_2_SO_4_ (Sigma-Aldrich, St. Quentin Fallavier, France, purity > 99%) were performed at various concentrations from 0.5 to 100 mol m^−3^. For each operating condition, concentrations of retentate and permeate solutions (*C_r_* and *C_p_*, respectively) were measured by conductimetry (conductimeter PC 5000 L Phenomenal, VWR, Radnor, US). The rejection rate *R* is calculated as follows:

(1) $$R=1-\frac{C_{p}}{C_{r}}$$

For each applied pressure, the permeation flux (*J_v_*) was measured by weighing the permeate stream during a given time.

Between each filtration, the setup was washed, and pure water was filtered to estimate the intrinsic hydraulic membrane permeability *L_p_* from the slope of the linear evolution of water flux with applied pressure *J_w_* = f(Δ*P*).

(2) $$J_{w}=\frac{L_{p}}{\mu }\Delta P\;$$

With *μ* as the dynamic viscosity of the fluid.

## 3. Numerical Modelling

### 3.1. Transport Model

The porous medium is assumed to be one-dimensional, constituted of cylindrical and uniform pores. The flow is assumed to be laminar without temporal variation (steady state). Variations according to radial direction (potential, concentrations) are neglected and constant values, defined as the radial average, are considered in this model.

The transport of ionic solutes in the porous media is modelled from the fact that the molar flux satisfies the continuity equation and divergence of the flux $div({\vec{J}}_{i})$ is equal to zero. The transport of each solute (*J_i_*) in the pores is considered as the sum of three contributions induced by diffusion due to the gradient of chemical potential ($\vec{\nabla \mu_{i}})$, electro-migration due to the electrical gradient $\vec{\nabla \Psi }$ and convection [4]. In steady state, this equation is:

(3) $$div({\vec{J}}_{i})=div\left[-\frac{D_{i}}{RT}\left(\vec{\nabla \mu_{i}}+Fz_{i}\vec{\nabla \Psi }\right)+c_{i}\vec{V}\right]=0$$

where *D_i_*, *z_i_* and *c_i_* are the diffusivity, the valence and the local concentration inside pores of the solute *i*, respectively. *R* is the ideal gas constant, *T* is the temperature, *F* is the Faraday constant (96,487 C mol^−1^), $\vec{\nabla \Psi }$ is the gradient of electric potential in the pore and *V* is the fluid velocity in the pore.

The influence of pore shape and tortuosity on the diffusive and convective transports is taken into account by introducing hindered factors *K_i,c_* and *K_i,d_* [26]. Equation (3) can be rewritten and is known as the extended Nernst–Planck equation:

(4) $$J_{i}=-K_{i,d}D_{i}\frac{dC_{i}}{dx}-K_{i,d}D_{i}c_{i}\frac{dln\gamma_{i}}{dx}-K_{i,d}D_{i}z_{i}c_{i}\frac{F}{RT}\frac{d\Psi }{dx}+K_{i,c}c_{i}V=VC_{i,p}$$

where *γ_i_* and *C_i,p_* are the activity coefficient (calculated by using the extended Debye–Hückel equation) and the permeate concentration of the solute *i*, respectively.

Hindrance factors for convection and diffusion are calculated by equations proposed by Bowen et al. [27]:

(5) $$K_{i,c}=\left(2-\phi \right)\left(1.0+0.054\lambda_{i}-0.988\lambda_{i}^{2}+0.441\lambda_{i}^{3}\right)$$

(6) $$K_{i,d}=1.0-2.3\lambda_{i}+1.154\lambda_{i}^{2}+0.224\lambda_{i}^{3}$$

where $\phi_{i}={\left(1-\lambda_{i}\right)}^{2}$ is the steric partitioning coefficient, which depends on *λ_i_*, which is defined as the ratio of the solute’s Stokes radius (*r_i,s_*) and pore radius (*r_p_*) λi=ri,srp [28].

The differential Equation (4) is solved for each solute *i* with two boundary conditions. The generalized chemical potentials of each solute at the interfaces between the bulk and the pore solution are equal at the pore inlet and outlet (both sides of the active layer x = 0, x = L). This equilibrium at the pore/solution interfaces can be described by the product of three contributions (steric, electric and dielectric), as proposed in Equation (5) [25].

The ratio between the concentration at the pore entry or outlet *c_i_*(*x*) and that of the feed or permeate solution *C_i_* (depending on the interface considered) can be calculated by:

(7) $$\mathrm{For}\;x=0\;\mathrm{or}\;L\;\frac{c_{i}\left(x\right)}{C_{i}}=\phi_{i}\frac{\gamma_{i,s}}{\gamma_{i,p}}\exp\left(-\Delta W_{i}\right)\exp\left(-\frac{z_{i}F}{RT}\Delta \Psi_{D}\right)$$

where $\Delta \psi_{D}$ is the Donnan potential (difference in electrical potential at both sides of the interface, free solution/solution in the pore) and $\Delta W_{i}$ is the solvation energy barrier due to the dielectric effect. This energy is computed in the model by considering an apparent dielectric permeability (ε_p_) of solution in the pore in the Born model [29]:

(8) $$\Delta W_{i}=\frac{\left(z_{i}e\right)}{8\pi \varepsilon_{0}r_{i,s}k_{B}T}\left(\frac{1}{\varepsilon_{p}}-\frac{1}{\varepsilon_{b}}\right)$$

where *e* is the electronic charge (*e* = 1.602 10–19 C), *k_B_* is the Bolzmann constant (*k_B_* = 1.381 10^−23^ m^2^ kg s^−2^ K^−1^), *ε_0_* is the permittivity of free space (*ε_0_* = 8.85419 10^−12^ F m^−1^) and *ε_b_* is the bulk dielectric constant (78.4 for water). It should be stressed that the impact of so-called “image forces” on dielectric exclusion, which is sometimes considered in Equation (7) [30,31], is not taken into account in this study.

For each ionic solute, Equations (4) and (7) are solved by respecting the electroneutrality condition in the feed solution (Equation (9)) and in the pore (Equation (10)):

(9) $$\overset{n}{\underset{i=1}{\sum }}z_{i}C_{i}=0$$

(10) $$\mathrm{for}\;0\;\leq \;x\;\leq \;L\;\sum_{i=1}^{n}z_{i}c_{i}\left(x\right)+X_{d}=0$$

where *X_d_* is the volumetric membrane charge density in the pore.

The resolution of this set of equations has already been detailed in a previous paper [32].

The permeation flux is calculated from Equation (11) by taking into account the osmotic pressure difference (Δπ) due to different salt concentrations on both sides of the membrane.

(11) $$J_{v}=\frac{L_{p}}{\mu }\left(\Delta P-\Delta \pi \right)$$

As the permeate concentration appears in the differential mass transfer equation (Equation (4)), the set of equations is iteratively solved by using an explicit Euler method up to convergence.

Four physical parameters are required (i.e., *L_p_*, *r_p_*, *X_d_* and *ε_p_*) to implement simulations. First, the hydraulic permeability *L_p_* was calculated from pure water filtration (Equation (2)). The mean pore radius is obtained from the filtration of a neutral solute (vitamin B12, *r_i,s_* = 0.72 nm and *D_i_* = 3.4 × 10^−10^ m^2^ s^−1^) by adjusting its value to fit experimental curve *R_i_* = f(*J_v_*). The mean pore radius is thus assessed by minimizing the quadratic error between experimental rejections and those calculated by a pore flow model based on the previously presented model, for which electro-migration, electric and dielectric contributions are neglected.

(12) $$R_{i}=1-\frac{\phi_{i}K_{i,c}}{1-\left(1-\phi_{i}K_{i,c}\right)exp\left(-\frac{K_{i,c}r_{p}^{2}\Delta P}{8\mu K_{i,d}D_{i}}\right)}$$

### 3.2. Adsorption Models

After being assessed, the volumetric membrane charge densities are normalised by feed salt concentration *X_d_*/*C_f_*, and the evolution of this normalised charge is linked to concentration by various adsorption isotherms [33].

- Freundlich isotherm, which empirically describes multilayer adsorption without saturation:

(13) $$\frac{X_{d}}{C_{f}}=K_{F}C_{f}{}^{\frac{1-n}{n}}$$

- Langmuir isotherm, which is a physical equation describing monolayer adsorption with surface saturation:

(14) $$\frac{X_{d}}{C_{f}}=\frac{Q_{max}K_{L}}{1+K_{L}C_{f}}$$

- Langmuir–Freundlich isotherm (also called the Sips isotherm), which is a hybrid equation combining the two previous approaches:

(15) $$\frac{X_{d}}{C_{f}}=\frac{Q_{max}K_{LF}C_{f}{}^{\frac{1-n}{n}}}{1+K_{LF}C_{f}{}^{\frac{1}{n}}}$$

In Equations (13)–(15), C_f_ represents the equilibrium feed concentration, 1/n the adsorption intensity and *Q_max_* the maximum adsorption capacity. *K_L_*, *K_F_* and *K_LF_* are the Langmuir, Freundlich and Langmuir–Freundlich constants, which are all related to adsorption capacity.

It should be noted that the Langmuir–Freundlich model is usually more suitable for predicting adsorption on heterogeneous surfaces, avoiding the limitation of the two other isotherms. For this reason, such a hybrid model is more adequate for mineral membranes which exhibit various kind of porosities.

Hence, at a low adsorbate concentration this model can be equated to the Freundlich model, and at a high concentration, it can predict monolayer adsorption, similar to Langmuir model.

## 4. Results and Discussion

### 4.1. Preliminary Results

Before investigating electric and dielectric exclusion mechanisms, the intrinsic hydraulic permeability of the various studied membranes was assessed from water flux measurements. The hydraulic permeability was estimated between each experiment to ensure that the structural properties of the membranes do not vary during the experimental campaign. Additionally, filtration of the vitamin B12 solution was performed to estimate the sieving properties of the membrane via the calculation of the mean pore radius with Equation (11). The values obtained for each membrane are summarised in Table 1.

Structural properties given in Table 1 show that the three membranes are notably different in both terms of permeability and pore size, but with different behaviours. The TiO_2_ membrane appears to be very close to nanofiltration with a tight pore size, whereas the Na-mordenite exhibits the largest pore size, typical of an ultrafiltration membrane. The MFI–MFI membrane shows intermediate pore size close to low MWCO UF membranes. Concerning hydraulic permeability, the trend is completely different and the TiO_2_ membrane demonstrates the highest permeability compared to the two others. This behaviour is probably due to a thinner skin layer of the TiO_2_ membrane. The steric exclusion is thus clearly different between these three membranes.

It should be noted that the experimental error on permeability is very low, less than 4% of variation between all the experiments. The *r_p_* value is estimated by fitting a rejection curve with a pore flow model, so error is mainly due to model assumptions. The experimental variation in VB12 rejection is about 5%. The corresponding variation is less than 10% in pore radius except for the Na-mordenite membrane. However, steric effects are negligible with this membrane due to large pores, so uncertainties do not have any impact.

### 4.2. Numerical Procedure

**Figure 1:**
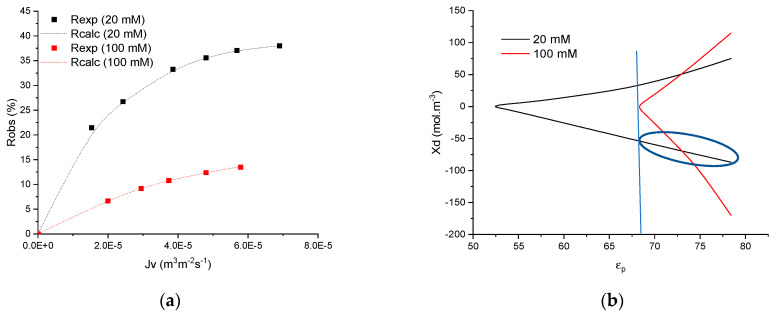
(**a**) Rejection curves of NaF obtained with TiO_2_ membrane for two feed concentrations (20 and 100 mol m^−3^). Symbols: experimental values and lines: simulated curves obtained with the various best-fitted parameters (*X_d_* and *ε_p_*) plotted in (**b**).

Once these two parameters are known, the model only required two other parameters, namely the dielectric constant of the solution confined in nanopores (*ε_p_*) and the volumetric membrane charge density (*X_d_*), which can be adjusted to fit experimental curves. However, it has already been highlighted that there exists and infinite number of couples (*ε_p_*, *X_d_*) which allow the description of one rejection curve [22,34], as it is represented for two NaF solutions with different concentrations in Figure 1a,b.

Figure 1a shows experimental and simulated rejection curves obtained by filtration of two solutions containing 20 and 100 mol m^−3^ of NaF. The simulated curves were obtained by assessing the various couples (*ε_p_*, *X_d_*) and the corresponding couples are drawn in a *X_d_* = f(*ε_p_*) graph (Figure 1b). From such curves, it is impossible to obtain the relevant couple that describes the experimental rejection curve. For this reason, physical simplifications are required.

In the case of 1:1 monovalent salts, an increase in membrane charge, either in negative or positive values, leads to an increase in rejection, the curve depicting the possible couples is always symmetrical on the *X_d_* = 0 axis.

First, zeta potential measurements were implemented on powders (synthesized in the same way than the active layers of the three membranes) during previous studies. The values do not have any importance for this study, but they can be found in references [24] for MFI, [25] for mordenite and [35] for TiO_2_. Indeed, only the sign of the membrane charge is required for physical simplification. In these references, it has been shown that the three membranes always exhibit a negative charge at natural pH, irrespective of the filtered solution considered. From this finding, only the negative part of the curve can be considered in Figure 1b.

The dielectric exclusion is induced by a change in the dielectric constant of the solution within membrane pores due to confinement. This decrease in the dielectric constant of the confined solution is mainly imputable to the orientation of solvent molecules due to the electric field induced by membrane charge and ions. The maximum dielectric exclusion thus necessarily corresponds to the highest concentration and the value of the dielectric constant estimated at the highest concentration is the lower limit, irrespective of the concentration of the solution. This deduction allows an estimation of the possible values for the dielectric constant of the solution. The dielectric constant cannot be lower than the minimum value obtained with the highest concentration. An example of the procedure is illustrated in Figure 1b. Considering the curves of potential (*ε_p_*, *X_d_*) obtained for 100 mol m^−3^ (curve in red), the minimum possible value of *ε_p_* is 69, which means that the value cannot be lower irrespective of the concentration considered. Additionally, given the physical meaning of the dielectric constant, it is obvious that its value cannot be higher than that of unconfined water, i.e., 78.4.

It should be stressed that the minimum value of the *ε_p_* range does not vary significantly between 75 (sometimes 50) and 100 mol m^−3^. Hence, there is no interest in increasing concentration above 100 mol m^−3^. For this reason, concentration was varied up to 100 mol m^−3^ and the minimum value of the dielectric constant is always identified at this concentration.

In this example, the apparent dielectric constant is thus in the range of 68 to 78.4 for all the NaF solutions filtered with the titania membrane. Using this range of *ε_p_*, the corresponding values of volumetric membrane charge density estimated to fit the experimental curve obtained with the solution of NaF 20 mol m^−3^ are between −69 and −87 eq m^−3^ (surrounded by the blue line in Figure 1b).

Once the intervals of normalised membrane charge (*X_d_*/*C_f_*) are determined for all the concentrations, the latter are then linked to concentration through adsorption isotherms. In this study, three usual isotherms (Langmuir, Freundlich and Langmuir–Freundlich) have been investigated, but it was found that two-parameter models (Langmuir or Freundlich) do not allow a correct description in some cases. Oppositely, the hybrid three-parameter model always led to a correct description of membrane charge evolution. The parameters of the isotherms were assessed by minimizing the quadratic criteria between the predicted *X_d_*/*C_f_*-value and the minimum and maximum values of the *X_d_*/*C_f_* range.

**Figure 2:**
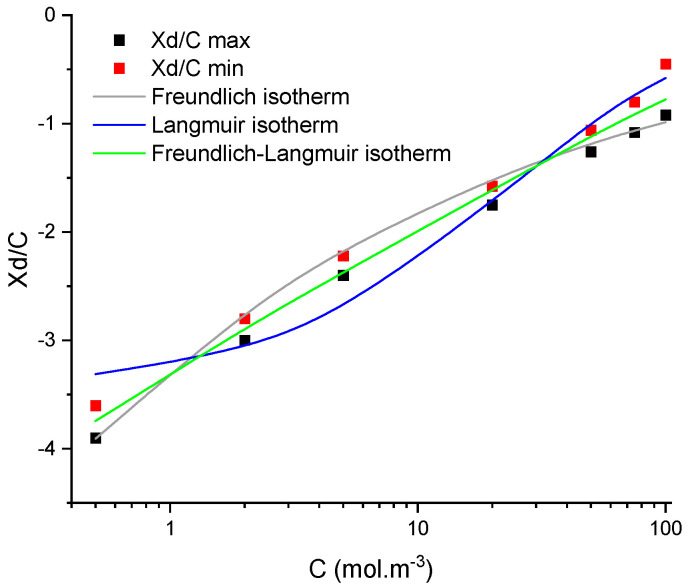
Evolution of minimum and maximum normalised volumetric charge densities with concentration obtained by fitting rejection of NaCl by TiO_2_ membrane and the corresponding best-fitted adsorption isotherms.

An example of the description by the three adsorption models is provided in Figure 2. It can be seen that the best description is obtained by the Freundlich–Langmuir model and it was thus chosen to use only the Langmuir–Freundlich isotherm to describe the experimental trends presented in the next section.

### 4.3. Numerical Investigation

**Figure 3:**
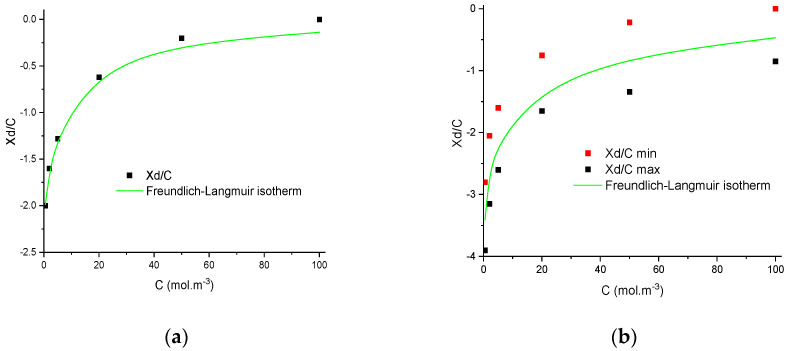
Evolution of minimum and maximum normalised volumetric charge density with concentration and the corresponding best-fitted Langmuir–Freundlich isotherms obtained by filtration of (**a**) NaI by the TiO_2_ membrane and (**b**) NaF by the Na-mordenite membrane.

The procedure presented in the previous section was implemented with various salts containing halide and sulfate ions at various concentrations and for three ceramic membranes. The experimental rejection rates were numerically fitted to obtain the curves (*X_d_*, *ε_p_*) relative to each salt concentration and each membrane. By assuming the assumptions previously reported, the range of the electric charge in the pore is estimated for each filtration, leading to minimum and maximum values of *X_d_*/*C_f_*. Finally, the best-fitted Langmuir–Freundlich isotherm is determined, and two examples are provided in Figure 3 for the filtration of iodide sodium by TiO_2_ membrane and sodium fluoride by the Na-mordenite membrane.

From Figure 3a,b, it can be seen that the interval of *X_d_*/*C_f_* for each concentration can strongly vary depending on the salt and the membrane considered. For instance, in the case of iodide sodium by the TiO_2_ membrane, the proposed method is very precise, and the range of possible *X_d_*/*C_f_* is only a point. Oppositely, with fluoride and Na-mordenite, a larger interval of value was obtained. In all the cases, the experimental data are correctly described by the Langmuir–Freundlich isotherms and the possibility of using these isotherms instead of the adjustable parameter *X_d_* for predictive purposes is investigated hereafter. It should be stressed that the dielectric constant considered for simulations was the mean value between the maximum and minimum values.

The fitted parameters of the Langmuir–Freundlich isotherms obtained with the various salts and membranes are given in Table 2.

**Figure 4:**
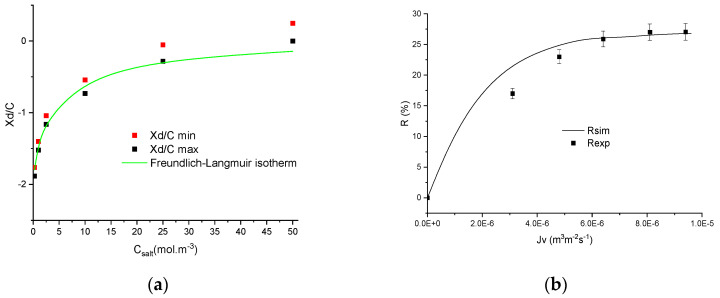
(**a**) Evolution of minimum and maximum normalised volumetric charge density with concentration and (**b**) rejection curve obtained experimentally and simulated from the corresponding best-fitted Langmuir–Freundlich isotherms obtained by filtration of Na_2_SO_4_ 0.44 mol m^−3^ by the MFI–MFI membrane.

**Figure 5:**
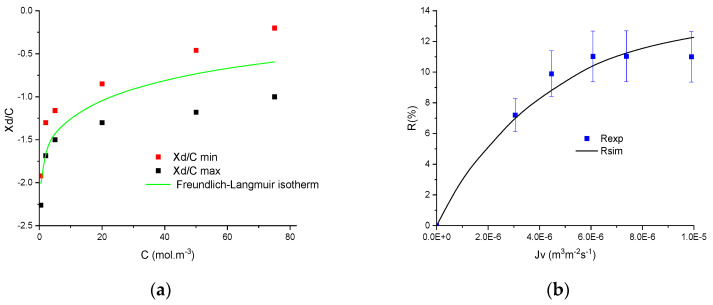
(**a**) Evolution of minimum and maximum normalised volumetric charge density with concentration and (**b**) rejection curve obtained experimentally and simulated from the corresponding best-fitted Langmuir–Freundlich isotherms obtained by filtration of NaI 2 mol m^−3^ by the MFI–MFI membrane.

**Figure 6:**
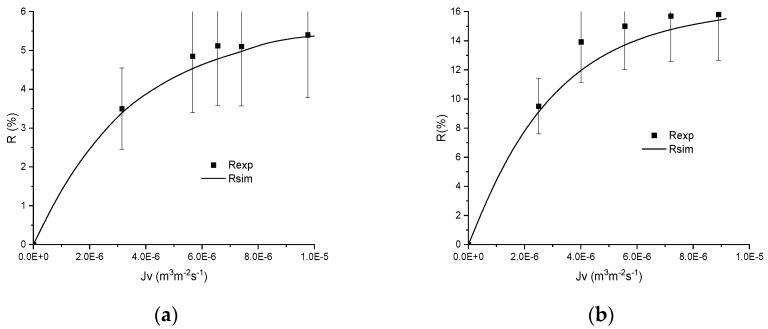
Rejection curves obtained experimentally and simulated from the corresponding best-fitted Langmuir–Freundlich isotherms obtained by filtration by the MFI–MFI membrane of NaF (**a**) 50 mol m^−3^ and (**b**) 2 mol m^−3^.

The ranges of *ε_p_* and *X_d_*/*C_f_* obtained with the various salts and membranes are summarised in Table 3 and examples are illustrated in Figure 4, Figure 5 and Figure 6.

A first example of experimental adsorption isotherm for Na_2_SO_4_ filtered by an MFI–MFI membrane is provided in Figure 4a and the simulations are compared with an experimental rejection curve obtained with 0.44 mol m^−3^ of Na_2_SO_4_ in Figure 4b.

It should be mentioned that the validation is carried out at concentrations used for the determination of adsorption isotherms. However, the parameters *ε_p_* and *X_d_*/*C_f_* were not assessed at this concentration but by using adsorption isotherms estimated with the whole range of *X_d_*/*C_f_* (between min and max values) for all the experimental data and the mean value of the dielectric constant estimated from all the experimental curves. This allows for discussion of the reliability of the novel procedure based on an adsorption isotherm and a mean value of dielectric constants estimated from squeezed ranges of values.

From Figure 4, it is highlighted that the simulated curve is in good agreement with the experimental curve. It should be noted that the isotherm does not correctly describe the normalised membrane charge *X_d_*/*C_f_* at high concentration due to the possible positive value, which is not possible to describe with the adsorption isotherm.

Figure 5 shows another example of procedure relevance and it is highlighted from this figure that the performance obtained with the filtration of a 2 mol m^−3^ NaI solution is correctly predicted by the model and the Langmuir–Freundlich isotherm without adjusting parameters.

Finally, the quality of the procedure was studied on two concentrations of NaF using the MFI–MFI membrane. The adsorption isotherm correctly describes the evolution of *X_d_*/*C_f_* and its use in simulations allows a suitable description of the experimental curves obtained by filtering 2 and 50 mol m^−3^ NaF solutions, as can be seen in Figure 6.

## 5. Conclusions

It is well-known that the difficulty in modelling and predicting membrane filtration performances lies in the assessment of model parameters. The main objective of this study was to develop a numerical tool able to estimate the salt rejection from adsorption isotherms using a novel procedure based on physical simplifications. First, this novel numerical procedure was proposed to estimate a range of reasonable values for the two main parameters, namely the dielectric constant of the confined solution and the volumetric membrane charge. Various adsorption isotherms have been investigated in this study and it was found that only the hybrid Langmuir–Freundlich isotherm allowed a correct description of all the experimental curves for heterogenous mineral membranes. This procedure was applied to various sodium salts and three mineral membranes, and the results highlighted that the experimental performances are correctly predictable with the proposed approach without adjusting parameters on the specific experimental condition.

## Figures and Tables

**Table 1:** Structural properties (*L_p_* and *r_p_*) and maximum VB12 rejection of the studied membranes.

Membranes	TiO_2_	Na-Mordenite	MFI–MFI
*L_p_* × 10^14^ (m^3^ m^−2^)	4.8	1.3	0.8
*R_VB12_* max (%)	62	3	36
*r_p_* (nm)	1.45	8.5	2.15

**Table 2:** Parameters of the Langmuir–Freundlich isotherms obtained with the various salts and membranes.

Membranes	Salts	*Q_max_*	*K_LF_*	*n*
TiO_2_	NaCl	3.37	1.20	0.022
	NaI	2.13	1.10	0.151
	Na_2_SO_4_	1.12	1.05	0.057
Na-MOR	NaCl	2.01	1.29	−0.010
	NaF	3.19	1.15	0.051
MFI/MFI	NaI	2.13	1.00	0.102
	NaF	1.26	1.10	0.050
	Na_2_SO_4_	1.87	0.99	0.274

**Table 3:** Minimum and maximum values of *ε_p_* and *X_d_*/*C_f_* estimated with the various salts and membranes.

Membranes	Salts	*ε_p_* min	*ε_p_* max	*X_d_*/*C_f_* min	*X_d_*/*C_f_* max
TiO_2_	NaCl	77.6	78.4	−3.8	0
	NaI	78.4	78.4	−2.0	0
	Na_2_SO_4_	77.8	78.4	−1.0	+0.36
Na-MOR	NaCl	77.6	78.4	−2.3	0
	NaF	73.3	78.4	−3.9	0
MFI/MFI	NaI	77.6	78.4	−2.3	0
	NaF	77.7	78.4	−1.3	0
	Na_2_SO_4_	77.9	78.4	−1.9	+0.25

## Data Availability

Data are available on request.

## Glossary

*c_i_*	Concentration of ion i within the pore (mol m^−3^)
*C_i,p_*	Permeate concentration of ion i (mol m^−3^)
*C_i,f_*	Feed concentration of ion i (mol m^−3^)
*D_i,__∞_*	Diffusion coefficient of ion i at infinite dilution (m^2^ s^−1^)
*e*	Electronic charge (1.602 10^−19^ C)
*F*	Faraday constant (96487 C mol^−1^)
*j_i_*	Flux of ion i (mol m^−2^ s^−1^)
*J_v_*	Permeation flux (m^3^ m^−2^ s^−1^)
*J_w_*	Permeation flux of pure water (m^3^ m^−2^ s^−1^)
*k_B_*	Boltzmann constant (1.381 10–23 m^2^ kg s^−2^ K^−1^)
*K_i,c_*	Ionic hindrance factor for convection (dimensionless)
*K_i,d_*	Ionic hindrance factor for diffusion (dimensionless)
*L_p_*	Hydraulic permeability (m^3^ m^−2^)
*R*	Gas constant (8.314 J mol^−1^ K^−1^)
*r_i,s_*	Stokes radius of ion i (m)
*R_i_*	Observed rejection of ion i (dimensionless)
*r_p_*	Average pore radius (m)
*T*	Temperature (K)
*V*	Solvent velocity in the pore (m s^−1^)
*x*	Axial position within the pore (m)
*X_d_*	Membrane effective charge density in the pore (eq m^−3^)
*z_i_*	Valence of ion i (dimensionless)
•	Greek letters
*γ_i,p_*	Activity coefficient of ion i in the pore (dimensionless)
*γ_i,s_*	Activity coefficient of ion i in the solution side of the interface (dimensionless)
Δ*P*	Applied pressure (Pa)
Δ*W_i_*	Dielectric exclusion energy (J)
Δ*ψ_D_*	Donnan potential (V)
Δ*π*	Osmotic pressure difference (Pa)
*ε_0_*	Permittivity of free space (8.85419 × 10^−12^ F m^−1^)
ε_b_	Bulk dielectric constant (dimensionless)
*ε_p_*	Pore dielectric constant (dimensionless)
*μ*	Dynamic viscosity (Pa s)
*φ_i_*	Steric partition coefficient (dimensionless)
*ψ*	Electrical potential within the pore (V)
